# Pain, Emotional Allodynia, and Relational Load (PEARL): Protocol of a Multicenter Observational Case–Control Study Validating the Emotional Allodynia Questionnaire in Fibromyalgia

**DOI:** 10.3390/jcm15187299

**Published:** 2026-09-19

**Authors:** Alberto Corriero, Mariateresa Giglio, Alfonso Pilolla, Paolo Trerotoli, Vittorio Schweiger, Enrico Polati, Maria Caterina Pace, Maria Beatrice Passavanti, Gabriele Finco, Salvatore Sardo, Boaz Gedaliahu Samolsky Dekel, Maria Cristina Sorella, Cinzia Di Venosa, Elisabetta Costantino, Daniela De Orsi, Filomena Puntillo

**Affiliations:** 1 Department of Interdisciplinary Medicine, Pain and ICU Section, University of Bari Aldo Moro, Piazza G. Cesare 11, 70124 Bari, Italy; alfonso.pilolla@gmail.com (A.P.); cinziadivenosa@gmail.com (C.D.V.); filomena.puntillo@uniba.it (F.P.); 2 Department of Interdisciplinary Medicine, Statistics Section, University of Bari Aldo Moro, Piazza G. Cesare 11, 70124 Bari, Italy; paolo.trerotoli@uniba.it; 3 Department of Surgery, Dentistry, Pediatrics and Gynecology, University of Verona, Piazzale L.A. Scuro 10, 37134 Verona, Italy; vittorio.schweiger@univr.it (V.S.); enrico.polati@univr.it (E.P.); 4 Department of Women, Child and General and Specialized Surgery, University of Campania “Luigi Vanvitelli”, 80138 Napoli, Italy; mariacaterina.pace@unicampania.it (M.C.P.); mariabeatrice.passavanti@unicampania.it (M.B.P.); 5 Department of Medical Sciences and Public Health, University of Cagliari, 09042 Cagliari, Italy; gabriele.finco@unica.it (G.F.); salvatore.sardo@unica.it (S.S.); 6 Anesthesia and Pain Therapy Unit, IRCCS Azienda Ospedaliero-Universitaria di Bologna, Policlinico S. Orsola-Malpighi, Via Massarenti 9, 40138 Bologna, Italy; boaz.samolskydekel@unibo.it (B.G.S.D.); mariacristina.sorella@unibo.it (M.C.S.); 7 Nutrimente Onlus, 20121 Milano, Italy; elisabettacostantinopsi@gmail.com; 8 Centro di Salute Mentale Area 5, ASL Bari, 70124 Bari, Italy; daniela.deorsi@asl.bari.it

**Keywords:** fibromyalgia, chronic pain, nociplastic pain, emotional allodynia, central sensitization, psychometrics, case–control studies, ROC analysis, phenotype

## Abstract

**Background**: The International Association for the Study of Pain defines pain as a sensory and an emotional experience, yet no instrument in routine practice quantifies affective hypersensitivity to low-intensity interpersonal stimuli, a pattern we term emotional allodynia, a construct transposed from thermal stimuli in major depression, which this study sets out to validate rather than assume. The Emotional Allodynia Questionnaire (AEQ), an 11-item self-report measure, was preliminarily validated in a single-center fibromyalgia sample, showing excellent internal consistency and a unidimensional structure. This protocol describes the multicenter validation phase. **Methods**: PEARL is a prospective, multicenter, observational case–control study at five Italian tertiary pain centers. Cases are adults with fibromyalgia diagnosed by the 2016 American College of Rheumatology (ACR) criteria; controls are adults with chronic non-cancer pain of defined nociceptive or neuropathic origin. All participants complete the AEQ together with the Central Sensitization Inventory (CSI), Pain Catastrophizing Scale, Beck Depression Inventory-II, State-Trait Anxiety Inventory, Difficulties in Emotion Regulation Scale and a pain visual analog scale, and are reassessed once at 40 ± 10 days. The targets are 274 cases and 151 evaluable controls, sized respectively for the precision of internal consistency within depression strata and for a 95% confidence interval of width 0.10 around an area under the curve of 0.80. Analyses cover internal structure, convergent and discriminant validity, test–retest reliability, AEQ–CSI phenotyping and covariate-adjusted discrimination. **Results**: Results are not yet available; recruitment began on 10 September 2025 and should be complete by approximately December 2027. **Conclusions**: The preliminary phase could not say whether the AEQ separates fibromyalgia from other chronic pain, whether the construct holds over time, or whether it survives adjustment for depression, anxiety, catastrophizing and emotion dysregulation. PEARL is designed to test these three questions, and to examine whether the AEQ–CSI phenotypes replicate. Registered on ClinicalTrials.gov (NCT07677631).

## 1. Introduction

The International Association for the Study of Pain (IASP) defines pain as both a sensory and an emotional experience [1], yet in routine pain practice the emotional dimension may not be assessed consistently, typically through indirect observation or general screening tools. These capture depression, anxiety, catastrophizing and broad emotion dysregulation, but none evaluates heightened affective sensitivity to interpersonal or relational stimuli.

This limitation is most evident in fibromyalgia, the prototypical nociplastic pain condition, marked by central amplification, symptom heterogeneity, and wide interindividual variability [2]. Some patients show marked distress in response to perceived exclusion, reduced reciprocity, or ambiguous social cues, while others, despite comparable pain, remain relatively resilient. We refer to this pattern as emotional allodynia (EA), by analogy with physical allodynia, in which normally innocuous stimuli provoke pain. The construct was first operationalized by Ushinsky and colleagues [3], who defined emotional allodynia as an altered negative response to normally non-aversive stimuli and demonstrated it with thermal testing in major depression, where it persisted regardless of the mood induced and so behaved as a trait rather than a state; we transpose it from thermal to relational stimuli, and from depression to fibromyalgia. Social rejection engages the anterior cingulate cortex, a region central to the affective dimension of physical pain [4]; neuroimaging has since shown that it also recruits the regions supporting the sensory component, namely the secondary somatosensory cortex and the dorsal posterior insula, so that social and physical pain share a pain-affect network [5]. The term is used by analogy, as in the report that introduced it. The IASP defines allodynia as pain due to a stimulus that does not normally provoke pain, and states that this is a clinical term that does not imply a mechanism [6]. What the present use borrows is that relation, between a normally non-aversive or low-intensity stimulus and a disproportionate aversive response. The analogy justifies the name; the construct rests on the psychometric properties examined in this phase. The Emotional Allodynia Questionnaire (AEQ) is, to our knowledge, the first operationalization of emotional allodynia in the relational domain. Table 1 sets emotional allodynia against the constructs nearest to it. The differences shown are operational, at the level of what each instrument scores and of the cue its items present; the empirical test of discriminant validity is specified in this protocol for emotion dysregulation and, by the present amendment, for pain catastrophizing. Alternative readings of the same clinical pattern are possible. The responses and the AEQ scores could reflect anxious anticipation of rejection or a general interpersonal sensitivity, neither of which the present battery measures. Emotional allodynia is therefore treated throughout as a construct under test, and Section 3 states the comparison with rejection sensitivity as the question this phase leaves open.

The AEQ was developed within the PEARL study to capture this dimension in a form brief enough for routine pain-clinic use; it is described in Section 2.5. The preliminary validation study [13] (*N* = 107 fibromyalgia patients) reported excellent internal consistency (Cronbach alpha 0.917, 95% confidence interval (CI) 0.880 to 0.943), a unidimensional exploratory factor structure (Kaiser–Meyer–Olkin measure (KMO) 0.90, loadings 0.55 to 0.86), partial confirmatory support, and moderate to strong convergent correlations with the Central Sensitization Inventory (CSI), Pain Catastrophizing Scale (PCS), Beck Depression Inventory-II (BDI-II), State-Trait Anxiety Inventory (STAI-Y) and Difficulties in Emotion Regulation Scale (DERS). Combining AEQ and CSI median splits yielded four exploratory profiles (Resilient, Emotional Allodynia, Central Sensitization, Mixed) that differed across all psychometric measures.

PEARL is a staged validation. The preliminary single-center phase established the internal structure, reliability and convergent validity of the AEQ in fibromyalgia. A subsequent analysis in an overlapping, expanded cohort (*n* = 136) examined which facets of emotion dysregulation account for the association [14]. Zero-order correlations between the AEQ and the DERS subscales ranged from 0.41 to 0.58; after simultaneous adjustment for depressive symptoms, trait anxiety, pain catastrophizing and central sensitization they fell to between 0.15 and 0.29. The reduction was not uniform. Difficulties with impulse control, access to regulation strategies, non-acceptance of emotional responses and emotional clarity remained associated with emotional allodynia; difficulty engaging in goal-directed behavior did not reach significance (*p* = 0.079), and lack of emotional awareness showed no association. Emotional allodynia therefore tracked difficulty in managing emotional states rather than inattention to them. An exploratory single-center network analysis of 149 patients [15] estimated where emotional allodynia sits among eleven other measures. Its correlation with depressive symptom severity (ρ = 0.502) fell to zero once the remaining measures were held constant, and was near zero when the penalty was removed (ρ = −0.033); its retained connectivity ran to emotion regulation (59.8%) and pain (30.2%) rather than to depression and anxiety (10.0%). Both analyses are exploratory and single-center, and neither settles the question; the overlap with pain catastrophizing was not resolved. They are reported because they set out what this phase has to test. This multicenter phase repeats the internal-consistency and factor analyses in the expanded cohort and, independently, in the cases recruited afterwards; adds a known-groups analysis against non-fibromyalgia chronic pain, for which no control participant has yet been enrolled and which the first phase was not designed to address; and assesses temporal stability.

The clinical rationale for the discriminative aim is that, if emotional allodynia is a relational-affective amplifier particularly recurrent in nociplastic pain [16], fibromyalgia patients should score higher than patients whose pain has an identifiable structural or neurological generator. Higher scores in fibromyalgia would not by themselves make emotional allodynia specific to that condition, since specificity would require comparison with the mixed and nociplastic presentations that the control group excludes by design.

The purpose of this phase is therefore to determine, in a multicenter sample, whether the internal consistency and the single-factor structure of the AEQ hold outside the setting in which they were first observed, and whether AEQ scores distinguish fibromyalgia from chronic pain of defined nociceptive or neuropathic origin.

## 2. Materials and Methods

### 2.1. Objectives

The primary objective is to confirm the internal consistency and factorial structure of the AEQ in the expanded multicenter fibromyalgia cohort, and in the independent subset of cases recruited after the preliminary analysis, and to estimate its known-groups accuracy in discriminating fibromyalgia from non-fibromyalgia chronic pain, expressed as the area under the receiver operating characteristic curve (AUC).

The secondary objectives are to estimate convergent validity against the comparator instruments described in Section 2.5; to examine discriminant validity with respect to the DERS and to the PCS; to estimate test–retest reliability in the full enrolled sample of both groups; to characterize floor and ceiling effects as indices of clinical acceptability; and to estimate internal consistency and convergent validity within the strata defined by the presence or absence of major depressive disorder, an analysis for which the cohort was sized. Three exploratory objectives complete the plan: deriving the AEQ–CSI median-split phenotypes within the cases and comparing their psychometric profiles, comparing those phenotypes with data-driven clustering, and estimating an exploratory AEQ cutoff for fibromyalgia versus non-fibromyalgia chronic pain. Each objective is operationalized as an endpoint in Section 2.6.

### 2.2. Study Design and Setting

PEARL (validation phase) is a prospective, multicenter, observational case–control psychometric validation study at five Italian tertiary pain centers, with no intervention. Under this protocol, cases and controls are enrolled consecutively and concurrently at each center. The baseline assessment is T0; all participants are reassessed once at T1, 40 ± 10 days later, an interval short enough to assume clinical stability of the construct yet long enough to limit recall of individual items. The Standard Protocol Items: Recommendations for Interventional Trials (SPIRIT) 2025 recommendations [17] were used as a completeness check on protocol content, adapted where items presuppose an allocated intervention; the populated checklist is Table S1 in the Supplementary Materials. Because PEARL is observational, reporting follows the Strengthening the Reporting of Observational Studies in Epidemiology (STROBE) statement [18].

The coordinating center is the Pain Outpatient Clinic of Policlinico Hospital, University of Bari Aldo Moro, Bari, Italy, where the PEARL study provides an established recruitment infrastructure and a pre-screened patient population. Participating centers are Italian tertiary pain units with active fibromyalgia and general chronic pain outpatient services, namely the University of Verona, the University of Campania Luigi Vanvitelli (Naples), the University of Cagliari, and the Policlinico S. Orsola-Malpighi (IRCCS Azienda Ospedaliero-Universitaria di Bologna). Enrollment at any center begins only after that center has its own ethics approval, or an extension of the coordinating-center approval, consistent with Italian regulatory requirements. At the time of writing this applies to the coordinating center alone: the PEARL study was approved for the Bari site on 8 September 2025, while the applications for the other four centers are under review.

The study is conducted in accordance with the ethical principles of the Declaration of Helsinki [19] and the IASP guidelines for pain research in humans [20].

### 2.3. Participants and Eligibility Criteria

Participants are enrolled consecutively; eligibility criteria are given in Table 2.

PEARL is a non-interventional study: no treatment is allocated, modified or withheld for study purposes, so there is no allocated intervention to discontinue, modify or adhere to. Concomitant care is unrestricted, and all pharmacological and non-pharmacological treatments continue according to standard clinical practice and the judgment of the treating clinician, recorded at baseline as descriptive and covariate information.

### 2.4. Registration, Ethics and Consent

The study is registered on ClinicalTrials.gov as an observational study (identifier NCT07677631; University of Bari Protocol Record PEARL-MC-2026; https://clinicaltrials.gov/study/NCT07677631 (accessed on 20 August 2026)), first posted on 1 July 2026. This protocol is version 2.1, dated 5 September 2026; the amendment revises the statistical analysis plan and does not affect eligibility, procedures, participant burden or consent. Enrollment began on 10 September 2025 at the coordinating center and is ongoing there; the participating centers and the control group start once the multicenter amendment has been approved locally. Recruitment should be complete by approximately December 2027. The sponsor is the University of Bari Aldo Moro (Piazza Giulio Cesare 11, 70124 Bari, Italy; contact: Alberto Corriero, alberto.corriero@gmail.com); there is no commercial sponsor.

The present multicenter amendment, with the updated informed consent forms, is submitted to the Local Ethics Committee of IRCCS Oncological Institute Gabriella Serio, IRCCS Giovanni Paolo II, Bari, Italy, which approved the PEARL study (Protocol 2451/CEL), and to the committees of the participating centers, for approval prior to multicenter enrollment. Any further protocol modification follows the same route: approval by the ethics committees before implementation, communication to the staff of every center and update of the registry record.

A trained investigator or designated research team member obtains written informed consent before enrollment. All participants have decision-making capacity and consent directly; refusal or withdrawal does not affect care.

No biological specimens are collected in this study. Participants consent to the use of their pseudonymized questionnaire and clinical data for the purposes of this validation study and for related methodological work within the PEARL study, and to being re-contacted once for the test–retest assessment. Apart from the contact details required for that single re-contact, which are stored separately and deleted after the retest window, no directly identifying information is retained in the research dataset.

### 2.5. Instruments and Procedures

Non-fibromyalgia chronic pain of defined nociceptive or neuropathic origin is the control condition, so that both groups live with chronic pain and pain-free status is not confounded with diagnosis. Test–retest reliability is assessed in both groups, because the temporal stability of the AEQ has to hold across chronic pain and not only within one diagnostic label. The mechanism is classified by the pain physician of the center on the clinical and instrumental documentation already available, and the classification, with the documentation supporting it, is recorded on the case report form. Patients whose pain cannot be attributed to a defined nociceptive or neuropathic generator, those with a mixed presentation, and those meeting criteria for a recognized nociplastic condition are not enrolled as controls.

The AEQ [13] consists of 11 items scored on a 0 to 4 Likert scale (0 = Never, 1 = Rarely, 2 = Sometimes, 3 = Often, 4 = Always) that assess the frequency of maladaptive emotional responses to subtle interpersonal or relational cues, such as delayed responses, unresponsiveness, and loss of reciprocity. The total score ranges from 0 to 44, with higher scores indicating greater affective hypersensitivity. The questionnaire was developed in Italian; the English version used for publication is an author’s translation. It is not administered to participants, and all data at every center are collected in Italian. Adaptation to another language is outside the scope of this phase. It would require forward and backward translation, review by an expert committee, cognitive debriefing and psychometric validation in the target population, and it is planned for after the properties of the Italian instrument have been established in the present multicenter sample. The full instrument, with its administration instructions and scoring, is published in the preliminary validation study [13].

The assessment battery comprises the AEQ; the CSI [22]; the PCS [12]; the BDI-II [23]; the STAI-Y, forms 1 and 2 [24]; the DERS [10], administered in the Italian 33-item adaptation [25]; and a 0 to 10 visual analog scale (VAS) for current pain intensity. A standardized self-report form also collects sociodemographic, clinical, lifestyle, and relational variables (age, sex, educational level, occupational status, marital status, living arrangement, pain duration, physical activity, sleep quality, perceived quality of life, relational history, and current or past psychological and pharmacological treatments). The estimated completion time is approximately 25 to 30 min.

The questionnaires are self-administered on paper, in the clinic, without assistance with item content. A physician is present and, on request, clarifies the meaning of an item without suggesting or influencing the response, and checks before the participant leaves that every item has been answered. The procedure is identical in the two groups and is the one used across the PEARL study, and the instruments are administered in a fixed order: the pain VAS, then the AEQ, followed by the PCS, the CSI, the BDI-II, the DERS and the two STAI-Y forms. The AEQ is placed first among the questionnaires: its responses are therefore not preceded by items on catastrophizing, mood, anxiety or emotion regulation, and it is not answered at the end of a half-hour battery. The order is not counterbalanced, so any order effect is constant across participants and carried equally by all analyses. These procedures are set out in a written standard operating procedure distributed to every participating center as part of its initiation pack, so that administration, the permitted scope of clarification and the completeness check are identical across centers.

### 2.6. Outcomes

Endpoints, with the measurement variable, the analysis metric and the time point for each, are given in Table 3.

### 2.7. Participant Timeline

The enrollment and assessment schedule is shown in Table 4; the main analyses use the baseline assessment.

### 2.8. Sample Size

Following the calculation reported in the preliminary study [13], 137 participants per stratum give a 95% confidence interval of 0.75 to 0.85 around a Cronbach alpha of 0.80 on 11 items, hence 274 fibromyalgia cases under approximately equal strata defined by major depressive disorder. Recruitment applies no quota: at the 46.3% depressive comorbidity recorded in the 149 cases enrolled by 8 July 2026 the smaller stratum is projected at about 127 rather than 137, which would require about 296 cases; the resulting confidence interval around alpha widens by less than 0.01, so the target is unchanged and the stratified analysis is secondary. The subset recruited after the preliminary analysis is expected to comprise about 125 cases, for which the interval around an alpha of 0.80 is 0.744 to 0.848, the precision of the main calculation.

The known-groups test (null AUC 0.70 versus 0.80, two-sided, alpha 0.05, 80% power) needs 97 evaluable participants per group, a number the cases already exceed, but leaves the area under the curve estimated only to within about 0.15; the control cohort is therefore sized for precision, a confidence interval of total width 0.10 requiring 151 evaluable controls. No dropout inflation is applied: only the retest is exposed to attrition. Computations used PASS 2024 (versions 24.0.5 and 24.0.9) [26] following the methods of Hanley and McNeil [27] and Obuchowski and McClish [28]. No formal power calculation was performed for the factor-analytic endpoints. Their adequacy depends on the communalities and on the overdetermination of the factor rather than on sample size alone [29], and the requirement falls as loadings increase [30]. The AEQ is a single factor with 11 indicators, and the preliminary study reported loadings of 0.55 to 0.86 with a KMO of 0.90. The target of 274 cases corresponds to 25 participants per item in the full cohort. The split-sample analyses activate at 200 cases, when each half gives about 9 participants per item, rising to about 12.5 per item at the full target. Fit indices estimated in halves of that size carry appreciable uncertainty, which is why the RMSEA is reported with its 90% confidence interval and both the unmodified and the modified models are reported.

### 2.9. Recruitment, Allocation and Bias Minimization

Participants are recruited consecutively from outpatient services at five Italian tertiary pain centers. Cases are identified from fibromyalgia services; controls are identified among patients attending the same services for chronic non-cancer pain of defined nociceptive or neuropathic origin. Every eligible patient presenting during the recruitment period is considered for enrollment, to limit selection bias. Enrollment is monitored centrally through the online data-collection platform. If a site falls below its enrollment target, protocol-compliant support measures are implemented in agreement with the Steering Committee. The estimated recruitment period across sites is 12 to 18 months. To document the representativeness of the sample and to support transparent reporting in accordance with the STROBE statement for observational studies [18], each center maintains a screening log recording all patients assessed for eligibility, the numbers eligible and enrolled, and the reasons for non-enrollment, summarized as a participant-flow diagram.

Selection bias is limited by consecutive sampling at each center, information bias by an administration procedure identical in the two groups and by the data-quality controls of Section 2.10. Comparability rests on the parallel eligibility criteria of Table 2; residual imbalance in age, sex, pain duration, depressive and panic comorbidity and concomitant treatments is handled by covariate adjustment and sensitivity analyses. Because the case group combines participants from the established PEARL cohort with cases newly recruited across the participating centers, recruiting center is recorded and examined as a potential source of heterogeneity, with a center-adjusted sensitivity analysis of the discriminative comparison where numbers permit.

Group membership is determined by the clinical diagnosis established before enrollment and is not assigned by the investigators, so there is no randomization, allocation sequence or concealment, and no one is blinded to diagnostic group. Scoring is automated within the data-collection system, data entry personnel are kept unaware of the study hypotheses where feasible, and the primary analyses follow the plan specified in this protocol. For the cases already enrolled before the plan was fixed, their data had been collected and the preliminary analyses examined, so the analyses repeated in that cohort are not prospectively pre-specified. For the cases recruited after the preliminary analysis, and for the control cohort, the plan preceded enrollment, and only the analyses in these cohorts are described as prospectively pre-specified. The known-groups comparison was specified before any control participant was enrolled, but it draws on cases accrued earlier, so its prospective status holds for the control cohort alone.

### 2.10. Data Collection and Management

The research team enters the paper responses into a secure online form with validation checks and mandatory fields for the primary variables. Range and logic checks at entry, and verification of a random sample of records against the source forms, guard against transcription error; residual missing items are handled as specified in Section 2.11. Diagnostic group, the defining pain mechanism for controls, depressive and panic comorbidity and concomitant treatments are recorded at baseline. Major depressive disorder and panic disorder are recorded as clinical diagnoses documented in the clinical record, current or past, ascertained as described in the note to Table 2; they are not derived from the BDI-II or STAI-Y scores.

All participants are telephoned once to schedule the retest in clinic at 40 ± 10 days; no further attempt, reminder or incentive is used. Those reassessed outside the window remain in the cross-sectional analyses but not in the test–retest analysis. The proportion actually reassessed, and the reasons for non-evaluable assessments, are reported.

The paper originals are retained as source documents. Access is role-stratified: clinical staff see only the instruments relevant to each assessment, the statistician the full pseudonymized dataset. The dataset is locked after all baseline and retest assessments and exported to R (version 4.3 or later).

The site principal investigators have access to the pseudonymized data of their own center. The coordinating investigator and the study statistician have access to the full pooled pseudonymized dataset for the analyses specified in this protocol. No contractual agreement limits the access of any investigator to the data. Requests for access from outside the study team are handled as described in the Data Availability Statement.

Personal data are processed under the General Data Protection Regulation (GDPR, EU Regulation 2016/679). Each center is the controller for its own patients’ personal data; the coordinating center is the controller for the pooled pseudonymized dataset. The processing of health data rests on explicit consent and on scientific research in the public interest (GDPR Article 9(2)(a) and 9(2)(j)). Each participant receives a study identifier at enrollment and only that identifier enters the dataset, which is therefore pseudonymized and not anonymized. The key is kept locally at each center, on paper in a locked cabinet or as an encrypted file, never online or shared; only pseudonymized data are pooled. The online form never contains directly identifying data and is hosted within the European Union by a provider under a GDPR-compliant data processing agreement. Contact details for the retest are stored separately and deleted at the end of the retest window. Data are retained for five years and then destroyed under institutional policy. Participants may exercise access, rectification and erasure through the local investigators or the Data Protection Officer, and may withdraw at any time, in which case their data are deleted. Data shared for publication are anonymized and aggregated.

### 2.11. Statistical Analysis

Descriptive statistics characterize the two groups. Demographic and clinical variables are reported as mean and standard deviation when approximately normally distributed; psychometric scale scores and VAS are reported as medians and interquartile ranges given their typically non-normal distribution. Categorical variables are reported as counts and percentages. Floor and ceiling effects for the AEQ are explored descriptively and reported if more than 15% of participants score at the lowest or highest possible value [31].

Internal consistency of the AEQ is assessed in the fibromyalgia cases using Cronbach alpha [32] and McDonald omega [33] with 95% confidence intervals; item-total correlations below 0.30 flag weak or redundant items. Exploratory factor analysis on item-level data uses minimum residual extraction with oblimin rotation, the KMO, the Bartlett test of sphericity and parallel analysis [34], with loadings of 0.30 or greater considered meaningful. A single-factor confirmatory model treats the 0 to 4 items as ordinal and is estimated by robust weighted least squares on the polychoric correlation matrix, with maximum likelihood and full information maximum likelihood for missing data as a sensitivity analysis, judged by comparative fit index (CFI) and Tucker–Lewis index (TLI) of 0.90 or greater, root mean square error of approximation (RMSEA) of 0.08 or less with 90% confidence interval, and standardized root mean square residual (SRMR) of 0.08 or less [35]. Residual covariances suggested by modification indices are added one at a time and only where the item pair is theoretically justified, and both the unmodified and the modified model are reported. Internal consistency and both factor analyses are computed in the full fibromyalgia cohort and repeated, with the same estimators and thresholds, in the subset of cases recruited after the preliminary analysis. Once 200 cases are available, the exploratory and the confirmatory analyses are conducted on separate random halves of the cohort, split once with a fixed seed, so that the confirmatory model is not fitted to the data that generated it.

The known-groups (discriminative) analysis is the primary inferential test of the validation phase. A ROC curve is constructed using the AEQ total score as the classifier and diagnostic group (fibromyalgia versus non-fibromyalgia chronic pain) as the reference, with fibromyalgia treated as the positive condition consistent with the hypothesis that higher AEQ scores indicate a higher probability of fibromyalgia. The AUC is estimated with its two-sided 95% confidence interval and tested against the null value of 0.70, the interval and the test being obtained by the method of DeLong. An optimal cutoff is identified using the Youden index [36], with accompanying sensitivity and specificity reported as hypothesis-generating. As a secondary discriminative analysis, the bidimensional AEQ–CSI combination is also evaluated, the two scores being combined by logistic regression and the predicted probability used as the classifier, the two curves being compared by the method for correlated ROC curves derived from the same cases [37]. To verify that discrimination is not driven by general affective burden or other potential confounders, a covariate-adjusted logistic-regression analysis is fitted in two steps: a base model adjusting for age, sex, and recruiting center; and an affect-adjusted model additionally including the established affective and pain-related comparators (BDI-II, STAI-Y, PCS, and DERS total scores, with major depressive and panic comorbidity recorded at baseline). The incremental discriminative contribution of the AEQ is quantified as the change in the area under the curve and the statistical significance of the AEQ term when added to these models. In a sensitivity analysis the affect-adjusted model is refitted with pain duration and concomitant treatments added, the latter coded as the presence or absence of each pharmacological class recorded at baseline. The apparent performance of every model fitted and evaluated in the same sample is corrected for optimism by bootstrap resampling. The distribution of control pain mechanisms is reported, and the area under the curve is estimated separately in the nociceptive and in the neuropathic control subgroups, each against the full case group, with 95% confidence intervals. These subgroup estimates are specified in the present amendment, before any control participant has been enrolled. They are exploratory, are not powered for a formal comparison between subgroups, and are reported descriptively.

Convergent validity is assessed by Spearman rank correlations between AEQ and CSI, PCS, BDI-II, STAI-Y, and DERS. Discriminant validity with respect to DERS is examined by a median-split cross-tabulation (chi-square test) to quantify dissociated profiles, and by comparing the average variance extracted of the AEQ, computed from standardized confirmatory loadings, with its squared correlation with the DERS total score. The same comparison is repeated in a two-factor measurement model in which the AEQ items and the six DERS subscales load on their respective factors, so that the average variance extracted of both constructs is set against the squared latent correlation, the form in which the Fornell–Larcker criterion [38] is defined. The same two comparisons are performed against the PCS, with its total score in the first and its three subscales as indicators of a latent factor in the second, since a preliminary network analysis identified pain catastrophizing, rather than emotion dysregulation or depressive symptom severity, as the measure whose separability from the AEQ those data could not resolve [15]. This comparison is added by the present amendment. The two versions can diverge, because the observed correlation is attenuated by measurement error; both are reported, and neither is treated as decisive on its own. The test–retest reliability of the AEQ total score is estimated in the full enrolled sample of both cases and controls using the intraclass correlation coefficient (two-way mixed model, absolute agreement) [39] with a 95% confidence interval, complemented by Bland–Altman inspection of agreement [40]. The same coefficient is estimated for the CSI, PCS and DERS total scores, which are re-administered at T1, so that the temporal stability of the AEQ is read against instruments of established reliability measured over the same interval. Estimates are reported for the pooled sample and separately for cases and controls. Participants who report a new treatment, a dose increase or a significant life event in the interval are identified from the interim clinical update and analyzed in a sensitivity analysis.

Phenotyping follows the preliminary approach: AEQ and CSI are dichotomized at their sample medians within the fibromyalgia cases (with values equal to the median classified in the higher category), generating four profiles (Resilient, Emotional Allodynia, Central Sensitization, Mixed). The preliminary medians (AEQ 16, CSI 61) are reported as reference values, while operative cutoffs are recomputed within the present cases. Between-phenotype differences on measures not used to define the phenotypes, which are tested by Kruskal–Wallis analysis followed by Dunn post hoc comparisons with Bonferroni correction [41], with epsilon-squared effect sizes. Given the statistical limitations of median splits, all phenotypic analyses are interpreted as exploratory. All tests are two-sided with significance set at 0.05. Because a single primary inferential test is specified, no global multiplicity correction is applied across the plan, beyond the Bonferroni adjustment specified above for the Dunn comparisons. The secondary and exploratory analyses are therefore interpreted through effect estimates and confidence intervals rather than through nominal significance, and any finding of interest requires replication in an independent sample.

Unsupervised clustering is conducted on the psychometric profile to examine whether data-driven structure recovers the theory-driven AEQ–CSI phenotypes. The primary model-based method, specified in the present amendment, is latent profile analysis, a Gaussian finite mixture fitted to the continuous scale scores with the number of profiles selected by the Bayesian information criterion, so that no categorization of the scores is required; k-means, hierarchical clustering and the tertile-coded latent class analysis used in the preliminary phase are reported alongside it, for continuity and for comparison of the solutions. Consistent with the preliminary finding that data-driven methods alone may not separate the Emotional Allodynia and Central Sensitization profiles, clustering is framed as support for, not replacement of, the theory-driven framework. The depression-stratified analysis is a secondary analysis recomputing internal consistency and convergent validity within the strata defined by the presence or absence of major depressive disorder. Statistical analyses are performed in R (version 4.3 or later) [42], with the psych [43], lavaan [44], pROC [45], irr [46], mclust and poLCA [47] packages as appropriate.

Because participants complete the questionnaires on paper with physician-verified completeness before leaving the clinic, item-level missing data are minimized by design and are expected to be rare. Should any residual missing item nonetheless occur (for example, an item missed at transcription and not recoverable from the source form), it is handled by pairwise deletion under the robust weighted least squares estimator and by full information maximum likelihood in the maximum-likelihood sensitivity model. All enrolled participants who meet the eligibility criteria of their group contribute to the cross-sectional analyses, in the diagnostic group recorded at baseline. Participants without a valid retest assessment are excluded only from the test–retest analysis. Total scores are computed only when every item of the scale is present, no prorating or item-level imputation being applied to the scores used in the score-based analyses. Complete-case results are reported alongside any model-based handling of missing data.

### 2.12. Study Oversight, Safety and Patient Involvement

The coordinating center at the Pain Clinic of Policlinico Hospital, Bari, oversees daily study management, including recruitment coordination, data entry oversight, and protocol adherence. The Steering Committee, composed of the principal investigator, the study statistician, and the site principal investigators, provides scientific oversight and reviews study milestones, meeting every three months or more frequently if required.

Given the observational, minimal-risk design, a formal data monitoring committee is not required and none is convened: there is no intervention to stop, no interim decision to take and no safety signal to adjudicate.

The only foreseeable burden is mild emotional discomfort while completing questionnaires on relational and affective experiences. A clinician is present and can offer a pause or refer the participant through the usual care pathways of the center; such episodes are documented on a standard form and reported with the results as counts and proportions by group and by center, together with any withdrawal that follows. Participation does not modify clinical care, and enrollment neither delays nor restricts any diagnostic or therapeutic decision, so no ancillary care or compensation scheme is foreseen.

Patients and the public were not co-investigators and did not select the outcomes or the instruments. The AEQ items, however, come from situations reported repeatedly by patients with fibromyalgia in consultation, and their wording was revised after the preliminary phase, whose completion times also gave the estimate of participant burden. Participants do not take part in recruitment or conduct. Results are returned to them in plain language through the participating clinics. The results will be published in a peer-reviewed journal regardless of the direction of the findings, and summary results will be posted on the ClinicalTrials.gov record.

## 3. Discussion

PEARL (validation phase) is the planned multicenter stage of the PEARL study, building directly on the preliminary single-center phase. It expands the fibromyalgia cohort across five centers and adds a newly recruited non-fibromyalgia chronic pain control group, thereby providing the first formal test of whether the AEQ discriminates fibromyalgia from other chronic pain conditions, and anchoring the size of the control cohort on this clinically interpretable known-groups objective.

Beyond replication, the central aim of this design is to test whether emotional allodynia is a distinct dimension rather than a restatement of established constructs. The combination of convergent validity, discriminant validity against emotion dysregulation by the Fornell–Larcker criterion [14,38], covariate-adjusted discrimination, and the depression-stratified analyses is intended to show that the AEQ captures variance not accounted for by depressive or anxious symptomatology, pain catastrophizing, or general difficulties in emotion regulation. Replication across multiple centers, and in particular in the cases recruited after the preliminary analysis, addresses whether the internal structure is generalizable rather than an artifact of a single setting, while the test–retest assessment tests the temporal stability expected of a trait-like characteristic suitable for stratifying patients.

Controls are drawn from patients with chronic pain rather than from pain-free individuals, so that any difference in AEQ scores cannot be attributed to living with pain. The direction of the hypothesis, that higher AEQ scores correspond to a higher probability of fibromyalgia, is consistent with the preliminary observation that emotional allodynia is particularly recurrent in this population.

A positive known-groups result would be open to a mechanistic reading at group level, while not constituting a direct measure of any single pain mechanism. Nociplastic pain is not synonymous with central sensitization: central sensitization is one of several mechanisms that commonly contribute to nociplastic pain, alongside altered descending modulation and affective-cognitive amplification [2,16]. Within the framework of nociplastic pain and the IASP definition that recognizes the affective component of pain [1], higher AEQ scores in fibromyalgia than in chronic pain sustained by a defined nociceptive or neuropathic generator would be consistent with emotional allodynia acting as a relational-affective amplifier of pain, plausibly distinct from and complementary to central sensitization as captured by the CSI. The partial independence of the AEQ and CSI dimensions, observed in the preliminary phase and modeled in the AEQ–CSI phenotyping, is consistent with their representing distinct contributing dimensions rather than a single underlying mechanism. In this sense the AEQ is intended to realign routine clinical assessment with the affective dimension of pain that conventional mood and cognitive instruments may capture only indirectly.

The study also strengthens the AEQ–CSI phenotyping framework that underpins subsequent interventional work in the PEARL study. Replicating the four-profile structure in the expanded multicenter cohort, and comparing it with data-driven clustering, tests whether the four profiles are recoverable without the median split, while clarifying the incremental value of the theory-driven model.

If these properties are confirmed, the AEQ and the AEQ–CSI phenotyping would offer a brief, infrastructure-free way of stratifying chronic pain by the dominant amplifying dimension, isolating a subgroup in whom relational-affective processes are more prominent and who might be studied for emotion-regulation-oriented care. That is the bridge to the interventional phase of the PEARL study, where treatments matched to a profile can be tested in prospectively identified subgroups instead of undifferentiated populations. Neither treatment-response heterogeneity nor clinical utility is evaluated in the present phase; both require the interventional study, and the phenotypes are therefore presented as a hypothesis for it rather than as a validated stratification tool.

Several limitations are acknowledged. The cases of the preliminary single-center publication are carried forward into the expanded cohort, so the internal-structure estimates obtained in the full cohort are not independent of it; this is precisely why the same analyses are pre-specified in the subset recruited afterwards, and why the known-groups comparison rests on a control group recruited de novo. The cross-sectional core design limits causal inference, although the test–retest assessment in the full enrolled sample of both groups addresses temporal stability. Control selection depends on the mechanistic classification of non-fibromyalgia pain and some misclassification is possible, mitigated by requiring a defined nociceptive or neuropathic generator and by excluding recognized nociplastic conditions from the control group. The median-split phenotyping retains the statistical limitations noted in the preliminary work; phenotyping, clustering and the cutoff remain exploratory, are reported without a global correction across them, and are interpreted as hypothesis-generating. The claim of this phase rests not on them but on the three primary endpoints of Table 3, internal consistency, factorial structure and known-groups accuracy, the last of which is its single hypothesis test, the AUC of the AEQ against a null value of 0.70. Because the two groups are defined to contrast mechanistically, the resulting area under the curve estimates known-groups validity rather than diagnostic performance in an unselected chronic pain population, where intermediate and mixed presentations are common. One of these limitations is not mitigated: rejection sensitivity has not been measured alongside the AEQ, so the operational separation set out in Table 1 remains untested, and the discriminant validity evidence of this phase, which addresses emotion dysregulation and pain catastrophizing, does not extend to it. A direct comparison is the priority for future validation.

## Figures and Tables

**Table 1:** Emotional allodynia and neighboring constructs.

Construct	What the Instrument Scores	What the Items Present as the Trigger	Difference from the AEQ
**Rejection sensitivity (Rejection Sensitivity Questionnaire, RSQ)** [7]	expected rejection weighted by anxious concern across 18 situations	hypothetical requests to significant others	anticipated rejection in hypothetical situations, not the frequency of a disproportionate response to cues that occurred; defined as an enduring disposition †
**Interpersonal sensitivity (Interpersonal Sensitivity Measure, IPSM)** [8]	“an undue and excessive awareness of, and sensitivity to, the behaviour and feelings of others”; five subscales	no specific cue; respondents rate how they are “generally”	a general disposition rather than a frequency of episodes; despite the “generally” instruction, IPSM scores fell after depressive improvement
**Negative mood (West Haven–Yale Multidimensional Pain Inventory, WHYMPI)** [9]	three items rating overall mood, irritability and tension over the past week	none; the items name no elicitor	recent affective burden, whereas the AEQ score is conditional on a specified weak interpersonal cue
**Emotion dysregulation (Difficulties in Emotion Regulation Scale, DERS)** [10]	difficulty regulating emotion once present: awareness, clarity, acceptance, impulse and goal control, strategies	distress already present; every item begins “When I’m upset”	that opening presupposes the emotion has arisen; the AEQ concerns the strength of the cue that precedes it
Illness invalidation (Illness Invalidation Inventory, 3 * I) [11]	discounting and lack of understanding by five social sources over the past year	illness-directed behavior of spouse, family, clinicians, work environment and social services	others’ conduct toward the patient, not the patient’s affect, and only in relation to the illness
**Pain catastrophizing (Pain Catastrophizing Scale, PCS)** [12]	13 items; “exaggerated negative orientation toward noxious stimuli”: rumination, magnification, helplessness	pain	elicitor and target are pain cognitions; discriminant validity against the PCS is a secondary endpoint of this phase (Section 2.6)

† Rejection sensitivity is described by its authors as an enduring disposition: the Rejection Sensitivity Questionnaire showed test–retest correlations of r = 0.83 at two to three weeks and r = 0.78 at four months.

**Table 2:** Eligibility criteria for fibromyalgia cases and for non-fibromyalgia chronic pain controls.

Criterion	Fibromyalgia Cases	Non-Fibromyalgia Chronic Pain Controls
**Inclusion**		
Age	18 years or older	18 years or older
Pain	Chronic non-cancer pain for at least 3 months	Chronic non-cancer pain for at least 3 months with a defined nociceptive or neuropathic generator ^a^
Diagnosis	Fibromyalgia, confirmed by the 2016 revision of the ACR criteria [21]	2016 ACR criteria for fibromyalgia not met
Language	Able to understand and independently complete self-report questionnaires in Italian	Able to understand and independently complete self-report questionnaires in Italian
Consent	Written informed consent	Written informed consent
**Exclusion**		
Oncological disease	Active oncological disease or cancer-related pain	Active oncological disease or cancer-related pain
Psychiatric history	Any current or past psychiatric diagnosis other than major depressive disorder and panic disorder ^b^	Any current or past psychiatric diagnosis other than major depressive disorder and panic disorder ^b^
Cognitive status	Severe cognitive impairment precluding questionnaire completion	Severe cognitive impairment precluding questionnaire completion
Language	Inability to understand or complete questionnaires in Italian	Inability to understand or complete questionnaires in Italian
Nociplastic condition	—	Fibromyalgia or another recognized nociplastic primary pain condition ^c^

ACR, American College of Rheumatology. ^a^ For example, radiologically or surgically documented degenerative, structural or post-surgical musculoskeletal pain, or neuropathic pain with an identifiable neurological lesion or disease. ^b^ Established from the clinical history, on a diagnosis made by a psychiatrist according to the current Diagnostic and Statistical Manual of Mental Disorders (DSM) criteria and recorded as such in the medical record; the procedure is identical at every center, as for any specialist diagnosis entered in the record. No structured research interview is administered. The exclusion was defined in consultation with a clinical psychiatrist, to avoid confounding from fundamental alterations in social cognition and emotional processing; major depressive disorder and panic disorder are instead recorded at baseline and used in the covariate-adjusted and stratified analyses of Section 2.11, to preserve ecological validity for an instrument intended for routine pain-clinic use. ^c^ For example primary chronic widespread pain [16]; excluded to keep the control group mechanistically distinct. Ongoing pharmacological treatment for pain or mental health is not an exclusion criterion in either group, since it represents standard of care.

**Table 3:** Primary, secondary and exploratory endpoints.

Endpoint	Measurement Variable	Analysis Metric	Time Point
**Primary**			
Internal consistency of the AEQ in fibromyalgia cases ^a^	AEQ item scores (0–4)	Cronbach alpha and McDonald omega, with 95% CI	T0
Factorial structure of the AEQ ^a^	AEQ item scores (0–4)	Exploratory factor analysis and single-factor confirmatory model (CFI, TLI, RMSEA, SRMR)	T0
Known-groups accuracy ^b^	AEQ total score (0–44) by diagnostic group	AUC with two-sided 95% CI, tested against a null value of 0.70	T0
**Secondary**			
Bidimensional AEQ–CSI discrimination	AEQ and CSI total scores by diagnostic group	AUC of the two-predictor combination, compared with the AEQ curve by the method for correlated ROC curves	T0
Covariate-adjusted discrimination	AEQ total score with age, sex, recruiting center and the affective and pain-related comparators	Change in AUC and significance of the AEQ term in logistic regression, base and affect-adjusted models	T0
Convergent validity	AEQ total score versus CSI, PCS, BDI-II, STAI-Y and DERS totals	Spearman rank correlation	T0
Discriminant validity with respect to the DERS and the PCS	AEQ, DERS and PCS total scores	Median-split cross-tabulation (chi-square) and average variance extracted versus squared correlation (Fornell–Larcker)	T0
Test–retest reliability	AEQ total score in cases and controls, with CSI, PCS and DERS totals as comparators	ICC, two-way mixed, absolute agreement, with 95% CI, and Bland–Altman inspection	T0 and T1
Floor and ceiling effects	AEQ total score	Proportion at the lowest and highest possible value, reported if above 15%	T0
Depression-stratified psychometrics	AEQ item and total scores within strata of major depressive disorder	Cronbach alpha and Spearman correlations within each stratum	T0
**Exploratory**			
AEQ–CSI phenotypes	AEQ and CSI totals dichotomized at the sample medians in cases	Four profiles compared on PCS, BDI-II, STAI-Y and DERS by Kruskal–Wallis and Dunn tests, with epsilon-squared	T0
Concordance of theory-driven and data-driven profiles	Psychometric profile of the cases	Latent profile analysis as the primary model-based method, with k-means, hierarchical clustering and latent class analysis reported alongside it, against the AEQ–CSI phenotypes	T0
Optimal AEQ cutoff	AEQ total score by diagnostic group	Youden index, with sensitivity and specificity, hypothesis-generating	T0

T0 and T1 are defined in Table 4. ^a^ Computed in the full fibromyalgia cohort and, separately, in the cases recruited after the preliminary analysis. ^b^ Also estimated separately in the nociceptive and in the neuropathic control subgroups, descriptively. CFI, comparative fit index; TLI, Tucker–Lewis index; RMSEA, root mean square error of approximation; SRMR, standardized root mean square residual; ICC, intraclass correlation coefficient; ROC, receiver operating characteristic.

**Table 4:** The schedule of enrollment and assessments. T0 is the baseline assessment; T1 is the single retest assessment, performed in all enrolled participants at 40 ± 10 days.

Procedure	Enrollment	T0 (Baseline)	T1 (Retest, All Participants, 40 ± 10 Days)
Eligibility screening (cases and controls)	X		
Informed consent	X		
Sociodemographic and clinical form		X	
Interim clinical update (new treatments, life events)			X
AEQ		X	X
CSI		X	X
PCS		X	X
BDI-II		X	
STAI-Y (1 and 2)		X	
DERS		X	X
VAS pain		X	X

X denotes a procedure performed at that time point. The AEQ, CSI, PCS, DERS and pain VAS are re-administered at T1; the BDI-II and the STAI-Y are not, since depressive and anxious symptomatology are baseline characteristics rather than retest targets. The interim clinical update records new treatments, dose increases and significant life events occurring in the interval, together with the time actually elapsed since T0.

## Data Availability

No datasets were generated or analyzed in the preparation of this protocol. The registered record of the study is publicly available at https://clinicaltrials.gov/study/NCT07677631 (accessed on 20 August 2026). The full protocol, including the statistical analysis plan, is this article, and is therefore publicly available on publication; the approved Italian version is available from the corresponding author on request. Participant-level data are not publicly accessible under Italian data protection legislation and the General Data Protection Regulation; the de-identified dataset supporting the planned analyses, together with the data dictionary and the statistical code, will be available from the corresponding author on reasonable request following publication of the main results.

## Supplementary Materials

The following supporting information can be downloaded at https://www.mdpi.com/article/10.3390/jcm15187299/s1, Table S1: The populated SPIRIT 2025 checklist for the PEARL protocol.

## Abbreviations

ACR, American College of Rheumatology; AEQ, Emotional Allodynia Questionnaire; AUC, area under the receiver operating characteristic curve; BDI-II, Beck Depression Inventory-II; CFI, comparative fit index; CI, confidence interval; CSI, Central Sensitization Inventory; DERS, Difficulties in Emotion Regulation Scale; DSM, Diagnostic and Statistical Manual of Mental Disorders; EA, emotional allodynia; GDPR, General Data Protection Regulation; IASP, International Association for the Study of Pain; ICC, intraclass correlation coefficient; IPSM, Interpersonal Sensitivity Measure; KMO, Kaiser–Meyer–Olkin; PCS, Pain Catastrophizing Scale; PEARL, Pain, Emotional Allodynia, and Relational Load; RMSEA, root mean square error of approximation; ROC, receiver operating characteristic; RSQ, Rejection Sensitivity Questionnaire; SPIRIT, Standard Protocol Items: Recommendations for Interventional Trials; SRMR, standardized root mean square residual; STAI-Y, State-Trait Anxiety Inventory, form Y; STROBE, Strengthening the Reporting of Observational Studies in Epidemiology; TLI, Tucker–Lewis index; VAS, visual analog scale; WHYMPI, West Haven–Yale Multidimensional Pain Inventory.
