# Supplementary material for: Pain, Emotional Allodynia, and Relational Load (PEARL): Protocol of a Multicenter Observational Case–Control Study Validating the Emotional Allodynia Questionnaire in Fibromyalgia
Source: J Clin Med. 2026 Sep 19;15(18):7299. doi: 10.3390/jcm15187299 (PMC13608470; doi:10.3390/jcm15187299)
Supplement: Supplementary file 1 [file jcm-15-07299-s001.zip › jcm-4551745-supplementary.pdf]

Study: Pain, Emotional Allodynia, and Relational Load (PEARL). Protocol version 2.1, 5 September 2026. ClinicalTrials.gov NCT07677631. PEARL is a non-interventional, prospective, multicenter observational case–control study; the SPIRIT 2025 items that presuppose an allocated intervention are reported as not applicable with the reason stated.

SPIRIT 2025 checklist of items to address in a randomized trial protocol*

Section / Topic	No	SPIRIT 2025 checklist item description	Reported on page no.
**Administrative information**			
Title and structured summary	1a	Title stating the trial design, population, and interventions, with identification as a protocol	Title, p. 1, line 2; article type "Study Protocol", line 1, p. 1, line 1. The title states the design (multicenter observational case–control study), the population (fibromyalgia) and the instrument under study, and identifies the paper as a protocol. PEARL is non-interventional, so no intervention is named.
	1b	Structured summary of trial design and methods, including items from the World Health Organization Trial Registration Data Set	Structured abstract (Background/Methods/Results/Conclusions), p. 1, line 26. The WHO Trial Registration Data Set items are held in the registry record NCT07677631, cited at the end of the abstract and in Section 2.4.
Protocol version	2	Version date and identifier	Section 2.4 (version 2.1, 5 September 2026), p. 6, line 207
Roles and responsibilities	3a	Names, affiliations, and roles of protocol contributors	Author list and affiliations, p. 1, line 5; Back matter, p. 16, line 618
	3b	Name and contact information for the trial sponsor	Section 2.4, sponsor and contact, p. 6, line 207
	3c	Role of trial sponsor and funders in design, conduct, analysis, and reporting of trial; including any authority over these activities	Section 2.4, p. 6, line 207; Back matter, p. 16, line 627; Back matter, p. 16, line 618. The sponsor is the University of Bari Aldo Moro, the academic institution of the coordinating center and of the investigators themselves; there is no commercial sponsor and the study receives no direct monetary funding. Sponsor and investigators therefore coincide: the design, conduct, analysis and reporting of the study are carried out by the investigators, as set out in the Author Contributions, and no external party holds authority over them.
	3d	Composition, roles, and responsibilities of the coordinating site, steering committee, endpoint adjudication committee, data management team, and other individuals or groups overseeing the trial, if applicable	Section 2.12, p. 14, line 518
**Open science**			
Trial registration	4	Name of trial registry, identifying number (with URL), and date of registration. If not yet registered, name of intended registry	Section 2.4, p. 6, line 207; end of abstract, p. 1, line 26. Registry, number, URL and date of registration (1 July 2026) are given. Enrollment began 10 September 2025, so registration was retrospective; this is stated explicitly.
Protocol and statistical analysis plan	5	Where the trial protocol and statistical analysis plan can be accessed	Back matter, p. 16, line 639. There is no separate statistical analysis plan document: the analysis plan is specified in full in Section 2.11, so the protocol and the analysis plan are this article and become publicly available on publication.
Data sharing	6	Where and how the individual de-identified participant data (including data dictionary), statistical code, and any other materials will be accessible	Back matter, p. 16, line 639; Section 2.10, p. 11, line 376

Section / Topic	No	SPIRIT 2025 checklist item description	Reported on page no.
Funding and conflicts of interest	7a	Sources of funding and other support (e.g., supply of drugs)	Back matter, p. 16, line 627
	7b	Financial and other conflicts of interest for principal investigators and steering committee members	Back matter, p. 16, line 650
Dissemination policy	8	Plans to communicate trial results to participants, healthcare professionals, the public, and other relevant groups (e.g., reporting in trial registry, plain language summary, publication)	Section 2.12, p. 14, line 533; Back matter, p. 16, line 639. Results are published regardless of the direction of the findings, summary results are posted on the ClinicalTrials.gov record, and results are returned to participants in plain language through the participating clinics; the full protocol, including the statistical analysis plan, is this article.
**Introduction**			
Background and rationale	9a	Scientific background and rationale, including summary of relevant studies (published and unpublished) examining benefits and harms for each intervention	Section 1, p. 2, line 54
	9b	Explanation for choice of comparator	Section 2.5, p. 7, line 234
Objectives	10	Specific objectives related to benefits and harms	Section 2.1, p. 4, line 142. PEARL administers no intervention, so the objectives concern measurement properties and known-groups accuracy rather than benefits and harms of a treatment; participant burden and distress are addressed in Section 2.12.
**Methods: Patient and public involvement, trial design**			
Patient and public involvement	11	Details of, or plans for, patient or public involvement in the design, conduct, and reporting of the trial	Section 2.12, p. 14, line 533
Trial design	12	Description of trial design including type of trial (e.g., parallel group, crossover), allocation ratio, and framework (e.g., superiority, equivalence, non-inferiority, exploratory)	Section 2.2, p. 5, line 160. Observational case–control design. Allocation ratio and trial framework (superiority, equivalence, non-inferiority) are not applicable because no intervention is allocated; the known-groups analysis tests the AUC against a null value of 0.70, as specified in Section 2.11.
**Methods: Participants, interventions, and outcomes**			
Trial setting	13	Settings (e.g., community, hospital) and locations (e.g., countries, sites) where the trial will be conducted	Section 2.2, p. 5, line 171
Eligibility criteria	14a	Eligibility criteria for participants	Section 2.3, p. 5, line 184; Table 2, p. 5, line 186
	14 b	If applicable, eligibility criteria for sites and for individuals who will deliver the interventions (e.g., surgeons, physiotherapists)	Section 2.2, eligibility of the participating centers, p. 5, line 171. No intervention is delivered, so there are no eligibility criteria for individuals delivering an intervention; the requirements for the personnel who administer and score the assessments are in Section 2.5.
Intervention and comparator	15a	Intervention and comparator with sufficient details to allow replication including how, when, and by whom they will be administered. If relevant, where additional materials describing the intervention and comparator (e.g., intervention manual) can be accessed	Not applicable: PEARL administers no intervention and no comparator. The exposure of interest is the diagnostic group, defined in Section 2.3, and the assessment battery given to both groups is described in full, with its administration procedure, in Section 2.5, p. 7, line 244.

Section / Topic	No	SPIRIT 2025 checklist item description	Reported on page no.
	15 b	Criteria for discontinuing or modifying allocated intervention/comparator for a trial participant (e.g., drug dose change in response to harms, participant request, or improving/worsening disease)	Not applicable: no intervention is allocated, so there is nothing to discontinue, modify or adhere to. Stated explicitly in Section 2.3, p. 6, line 201.
	15c	Strategies to improve adherence to intervention/comparator protocols, if applicable, and any procedures for monitoring adherence (e.g., drug tablet return, sessions attended)	Not applicable: no intervention is allocated, so there is nothing to discontinue, modify or adhere to. Stated explicitly in Section 2.3, p. 6, line 201. Completeness of the assessment itself is ensured by physician-verified completion at the point of care, described in Section 2.5 and Section 2.10.
	15 d	Concomitant care that is permitted or prohibited during the trial	Section 2.3, concomitant care unrestricted and recorded at baseline, p. 6, line 201
Outcomes	16	Primary and secondary outcomes, including the specific measurement variable (e.g., systolic blood pressure), analysis metric (e.g., change from baseline, final value, time to event), method of aggregation (e.g., median, proportion), and time point for each outcome	Section 2.6, p. 7, line 278; Table 3, p. 8, line 281. Table 3 gives, for every primary, secondary and exploratory endpoint, the measurement variable, the analysis metric and the time point.
Harms	17	How harms are defined and will be assessed (e.g., systematically, non-systematically)	Section 2.12, p. 14, line 526. Harms are assessed non-systematically: no intervention is administered and the only foreseeable harm is transient emotional discomfort while completing the questionnaires, documented whenever it occurs.
Participant timeline	18	Time schedule of enrollment, interventions (including any run-ins and washouts), assessments, and visits for participants. A schematic diagram is highly recommended (see Figure)	Section 2.7, p. 9, line 287; Table 4, schedule of enrollment and assessments, caption, p. 9, line 290
Sample size	19	How sample size was determined, including all assumptions supporting the sample size calculation	Section 2.8, p. 9, line 297
Recruitment	20	Strategies for achieving adequate participant enrollment to reach target sample size	Section 2.9, p. 10, line 325
**Methods: Assignment of interventions**			
Randomization:			
Sequence generation	21a	Who will generate the random allocation sequence and the method used	Not applicable: group membership is determined by the clinical diagnosis established before enrollment and is not assigned by the investigators, so there is no randomization and no allocation sequence. Stated explicitly in Section 2.9, p. 10, line 346.
	21 b	Type of randomization (simple or restricted) and details of any factors for stratification. To reduce predictability of a random sequence, other details of any planned restriction (e.g., blocking) should be provided in a separate document that is unavailable to those who enroll participants or assign interventions	Not applicable: group membership is determined by the clinical diagnosis established before enrollment and is not assigned by the investigators, so there is no randomization and no allocation sequence. Stated explicitly in Section 2.9, p. 10, line 346.
Allocation concealment mechanism	22	Mechanism used to implement the random allocation sequence (e.g., central computer/telephone; sequentially numbered, opaque, sealed containers), describing any steps to conceal the sequence until interventions are assigned	Not applicable: group membership is determined by the clinical diagnosis established before enrollment and is not assigned by the investigators, so there is no randomization and no allocation sequence. Stated explicitly in Section 2.9, p. 10, line 346.

Section / Topic	No	SPIRIT 2025 checklist item description	Reported on page no.
Implementation	23	Whether the personnel who will enroll and those who will assign participants to the interventions will have access to the random allocation sequence	Not applicable: group membership is determined by the clinical diagnosis established before enrollment and is not assigned by the investigators, so there is no randomization and no allocation sequence. Stated explicitly in Section 2.9, p. 10, line 346.
Blinding	24a	Who will be blinded after assignment to interventions (e.g., participants, care providers, outcome assessors, data analysts)	Section 2.9, p. 10, line 346. Participants and treating clinicians cannot be blinded to diagnostic group; the personnel responsible for scoring and data entry are kept unaware of the study hypotheses and scoring is automated within the data-collection system.
	24b	If blinded, how blinding will be achieved and description of the similarity of interventions	Not applicable: there is no intervention whose appearance could be matched. The masking that is meaningful here is described in Section 2.9, p. 10, line 346.
	24c	If blinded, circumstances under which unblinding is permissible, and procedure for revealing a participant's allocated intervention during the trial	Not applicable: no participant is allocated to an intervention, so no circumstance requires unblinding.
**Methods: Data collection, management, and analysis**			
Data collection methods	25a	Plans for assessment and collection of trial data, including any related processes to promote data quality (e.g., duplicate measurements, training of assessors) and a description of trial instruments (e.g., questionnaires, laboratory tests) along with their reliability and validity, if known. Reference to where data collection forms can be accessed, if not in the protocol	Section 2.10, p. 11, line 359; Section 2.5, assessment battery, p. 7, line 257. The AEQ, the instrument under evaluation, is summarized with its item count, response scale, score range and construct; the full instrument, with its administration instructions and scoring, is published in the preliminary validation study cited there. The comparator instruments are established measures cited to their source publications rather than re-described; the administration and data-quality procedures are identical for all of them.
	25b	Plans to promote participant retention and complete follow-up, including list of any outcome data to be collected for participants who discontinue or deviate from intervention protocols	Section 2.10, p. 11, line 368. The procedure is a single telephone call inviting the participant back to the clinic; no further attempt is foreseen, since the retest is one contact and not a follow-up programme. Participants who miss the window are documented and remain in the cross-sectional analyses.
Data management	26	Plans for data entry, coding, security, and storage, including any related processes to promote data quality (e.g., double data entry; range checks for data values). Reference to where details of data management procedures can be accessed, if not in the protocol	Section 2.10, p. 11, line 372; confidentiality, p. 11, line 381
Statistical methods	27a	Statistical methods used to compare groups for primary and secondary outcomes, including harms	Section 2.11, p. 11, line 396
	27b	Definition of who will be included in each analysis (e.g., all randomized participants), and in which group	Section 2.11, analysis populations, p. 13, line 505
	27c	How missing data will be handled in the analysis	Section 2.11, missing data, p. 13, line 505
	27d	Methods for any additional analyses (e.g., subgroup and sensitivity analyses)	Section 2.11, p. 13, line 491; covariate-adjusted and depression-stratified analyses also in Section 2.11.
**Methods: Monitoring**			
Data monitoring committee	28a	Composition of data monitoring committee (DMC); summary of its role and reporting structure; statement of whether it is independent from the sponsor and funder; conflicts of interest	Section 2.12, p. 14, line 523. No data monitoring committee is convened; the reason, an observational minimal-risk design with no intervention to stop, is given there.

Section / Topic	No	SPIRIT 2025 checklist item description	Reported on page no.
		and reference to where further details about its charter can be found, if not in the protocol. Alternatively, an explanation of why a DMC is not needed	
	28b	Explanation of any interim analyses and stopping guidelines, including who will have access to these interim results and make the final decision to terminate the trial	Not applicable: with no intervention and no allocation there is nothing to stop, so no interim analysis and no stopping rule is specified. The absence of interim decisions is stated in Section 2.12, p. 14, line 523.
Trial monitoring	29	Frequency and procedures for monitoring trial conduct. If there is no monitoring, give explanation	Monitoring of study conduct is not formal and no independent monitoring or audit body is described, there being no intervention, no investigational product and no procedure beyond questionnaire completion. The procedures that do apply are described in Section 2.10, p. 11, line 359 (validation checks and mandatory fields at entry, range and logic checks, and verification of a random sample of records against the source forms) and in Section 2.12, p. 14, line 518 (oversight of protocol adherence by the coordinating center, and review of study milestones by the Steering Committee every three months).
**Ethics**			
Research ethics approval	30	Plans for seeking research ethics committee/institutional review board approval	Section 2.4, p. 6, line 207; Back matter, p. 16, line 629; Section 2.2, p. 5, line 182
Protocol amendments	31	Plans for communicating important protocol modifications to relevant parties	Section 2.4, p. 6, line 217. Protocol modifications are approved by the ethics committees before implementation, communicated to the staff of every center and reflected in the registry record; the present multicenter amendment and the updated consent forms were submitted accordingly.
Consent or assent	32a	Who will obtain informed consent or assent from potential trial participants or authorized proxies, and how	Section 2.4, p. 6, line 224
	32b	Additional consent provisions for collection and use of participant data and biological specimens in ancillary studies, if applicable	Section 2.4, p. 6, line 227. No biological specimens are collected in this study or in any planned ancillary study; consent covers the use of pseudonymized questionnaire and clinical data within the PEARL study.
Confidentiality	33	How personal information about potential and enrolled participants will be collected, shared, and maintained in order to protect confidentiality before, during, and after the trial	Section 2.10, p. 11, line 381
Ancillary and post-trial care	34	Provisions, if any, for ancillary and post-trial care, and for compensation to those who suffer harm from trial participation	Section 2.12, p. 14, line 526. Mild emotional discomfort while completing the questionnaires is foreseeable and is managed within the visit: a clinician can offer a pause or refer the participant through the usual care pathways of the center, and the episode is documented and reported with the results. Participation does not modify clinical care and neither delays nor restricts any diagnostic or therapeutic decision; no ancillary care or compensation scheme is foreseen.

*We strongly recommend reading this checklist in conjunction with the SPIRIT 2025 Explanation and Elaboration and the SPIRIT 2025 Expanded Checklist for important clarifications on all the items. We also recommend reading relevant SPIRIT extensions. See www.consort-spirit.org

Citation: Chan A-W, Boutron I, Hopewell S, Moher D, Schulz KF, et al. SPIRIT 2025 statement: updated guideline for protocols of randomised trials. BMJ 2025;389:e081477. https://dx.doi.org/10.1136/bmj-2024-081477

© 2025 Chan A-W et al. This is an Open Access article distributed under the terms of the Creative Commons Attribution License (https://creativecommons.org/licenses/by/4.0/), which permits unrestricted use, distribution, and reproduction in any medium, provided the original work is properly cited.
